# Rural Post-Earthquake Resettlement Mode Choices: Empirical Case Studies of Sichuan, China

**DOI:** 10.3389/fpubh.2022.861497

**Published:** 2022-05-12

**Authors:** Lei Zhao, Sifan Zhou, Jinglin Zhong, Yibin Ao, Yan Wang, Tong Wang, Yunfeng Chen

**Affiliations:** ^1^College of Environment and Civil Engineering, Chengdu University of Technology, Chengdu, China; ^2^Department of Engineering Management, Sichuan College of Architectural Technology, Chengdu, China; ^3^Faculty of Architecture and the Built Environment, Delft University of Technology, Delft, Netherlands; ^4^School of Construction Management Technology, Purdue Polytechnic Institute, Purdue University, West Lafayette, IN, United States

**Keywords:** post-earthquake resettlement mode, influencing factor, factor analysis, binary logistic regression, Wenchuan earthquake, Changning earthquake, Lushan earthquake

## Abstract

Earthquakes occur frequently in rural areas of Sichuan, China, causing huge damage and high mortality. The built environment plays a significant role in providing residents with safe and resilient settlements in such areas. There is yet little research on how rural families in developing countries cope with geological disasters like earthquakes, and how built environmental factors would influence their resettlement choices which would directly affect their quality of life afterward. Urban planning activities should be accompanied by these insights to design and create human-centric resettlements accordingly. In this study, the resettlement choices after three major earthquakes in Sichuan were studied for this reason. Random sampling and face-to-face questionnaire surveys were combined with factor analysis and binary logistic regression to understand the resettlement modes desired by the residents and the influencing factors. The results show that residents who have lived in their current places long and whose houses were not built recently are more likely to choose the *in-situ* resettlement. Accessibility to employment and public services has a significant impact on residents' choice of *in-situ* resettlement or reallocated resettlement, and so does the previous resettlement experience. The research results can provide useful suggestions for Chinese rural area post-earthquake resettlement planning following a human-centric approach with empirical data.

## Introduction

**Figure 1:**
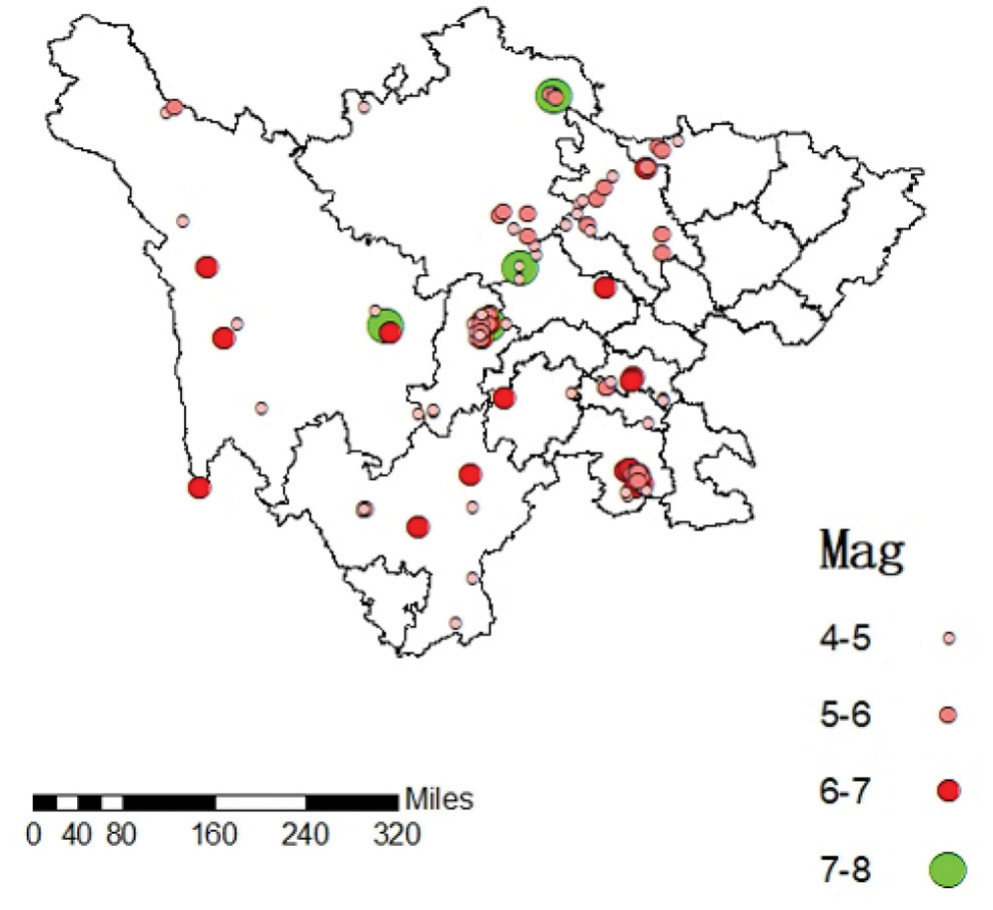
Earthquake distribution map of Sichuan region.

China is a country that suffers from severe earthquakes (1, 2). Sichuan is one of the regions that are prone to the most known earthquakes in the world (3). The earthquake distribution map of the Sichuan region is shown in Figure 1, from which it can be seen that earthquakes have occurred frequently in Yibin City, Ya'an City, and Aba Tibetan and Qiang Autonomous Prefecture for the past few years. This map is generated by ArcMap using Chinese seismological network data for earthquakes of magnitude 4 or above, from 2012 to 2020. The Wenchuan earthquake (in Aba Tibetan and Qiang Autonomous Prefecture) in 2008 caused the death of 69,227 people, 374,644 injured, and 17,923 missing; the Lushan earthquake (in Ya'an city) on April 20, 2013, killed 196 people, left 21 missing, and 11,470 injured; the Changning (in Yibin city) Earthquake on June 17, 2019, resulted in 11 deaths and 122 injuries (4). These earthquakes changed the natural environment of the disaster areas and left the environment there more disaster-prone and fragile. The residents are severely impacted by such disasters: their homes and farmland are buried and abandoned and their industries are relocated. They face the choice of resettlement, which directly affects their quality of life (5). Therefore, post-disaster resettlement planning for resilient rural areas for reducing disasters' impacts is particularly important for local residents (6). Post-earthquake resettlement planning shall follow the principles of putting people first, with scientific overall planning guidance and step-by-step implementations which combine self-reliance, state support, and social assistance (7).

The current research on resettlement choices after disasters mainly focuses on the relationship between residents' willingness to relocate and its influencing factors but lacks a systematic understanding of the choice made for specific post-disaster resettlement modes.

There are normally two modes for resettlement after a strong earthquake: *in-situ* resettlement and reallocated resettlement. *In-situ* resettlement refers to the resettlement of residents in their original place of residence (8). Reallocated resettlement refers to the relocation of residents to areas that are less prone to earthquakes. These two resettlement modes both have their unique advantages. *In-situ* resettlement does not require mobilization and land acquisition and therefore does not cause much social tension (8). Reallocated resettlement offers better employment, environmental and public services, and increases the chances of livelihood (9).

In addition, according to the existing research, post-disaster resettlement modes include centralized resettlement and scattered resettlement. Developing concentrated rural settlements in a village is a feasible way to achieve sustainable development and improve the resilience of villages after disasters. Shortcomings in scattered resettlement include wasting land resources, poor living conditions, and environmental degradation. Centralized resettlement is considered an effective means of utilizing rural land, improving infrastructure, public services, and rural living conditions (10, 11). However, in rural China, the scattered villages are the basic forms of villages (12). Therefore, it is still desired by some residents after earthquakes.

Furthermore, previous studies on post-disaster resettlement behavior mostly focused on developed countries (13–15), and on how urban families cope with hurricanes, floods, and other natural disasters (16–19). There is little research on how rural families in developing countries and poor areas cope with geological disasters like earthquakes. Knowing who is at higher risk due to their resettlement mode choices and the reasons behind their choices could help policymakers design targeted policies to reduce loss of life and damage for such understudied areas (20).

Recently, identifying impacting factors of farmers' resettlement choices has become a hot topic in academic research. Numerous studies show that the main factors affecting rural residents' choice of resettlement mode are on two levels: natural environment, resettlement planning, transportation conditions, government policies, and other macro factors; family structure, financial conditions, education level, disaster risk perception and other personal and family characteristics on the micro level (21). Studies have been conducted on the willingness of residents to move after disasters in the United States. The results show that the main migration groups caused by natural disasters are the elderly who have lived in a place for a short period of time and families with low education levels (22). Lu's research also finds that people who have lived in a place for years may not choose to move their jobs or homes elsewhere due to strong local dependency (23). An investigation into the migration of different populations after the 1970 Earthquake in Peru shows that many of the victims fell into poverty after the earthquake and many young people preferred to seek employment opportunities in big cities (24). The housing conditions also impact the willingness to move. Studies find that when the existing housing cannot meet the needs of residents, the willingness to move will increase (25). However, the longer the residents have lived in the local area, they become more dependent on the local area, leading to their reluctance to move. The appeal of a location increases with the accessibility to workplaces and everyday destinations (26). Therefore, convenient and diverse means of transportation will significantly affect residents' willingness to relocate. In the Chinese countryside, motorbikes are a good substitute for cars as farmers with motorbikes can easily reach the surrounding areas (27). Rural residents' satisfaction with the living environment also has an impact on rural residents' final choices. Studies show that people prefer to live in places that are comfortable, accessible, and free from disasters (28–31). Similarly, whether people will resettle is largely related to their risk perception of disasters (32, 33). Facing potential disaster risks, some people living in risky areas will decide to resettle, while others are reluctant to move. For rural residents who have experienced post-disaster resettlement, the experience of previous post-disaster resettlement is also an important factor affecting later choices (23). As illustrated, much research has been performed to identify and analyze impacting factors but these factors are not grouped under themes to be used for systematic analysis for a region in China, which makes it difficult for the local government to design specific resettlement options based on the preferences of the local residents.

To summarize, previous studies do not specify different modes of resettlement for rural residents after earthquakes and are mainly executed in developed countries for various impacting factors. It is needed to study rural residents' post-earthquake resettlement mode choices in developing countries like China as the rural residents suffer a lot from severe earthquakes and their quality of life should be improved with empirical studies for better planning. And it is also important to group influencing factors under themes to study each theme separately to holistically understand the impacts. Therefore, the purpose of this study is to explore the impact of factors under different themes on residents' choice of resettlement modes in Chinese rural earthquake-stricken areas. The rural residents affected by the three major earthquakes in Sichuan are contacted to study their post-disaster resettlement mode choices and influencing factors. Two groups of four post-disaster resettlement modes ([*in-situ* resettlement, reallocated resettlement] and [centralized resettlement, scattered resettlement]) are set up. The differences in residents' willingness to choose resettlement mode between the two groups after the earthquake have been compared. The results will shed light on further resettlement planning practices after earthquakes in the Chinese rural areas.

The following chapters are organized as follows: Chapter 2 explains the case areas and the identified impacting factor themes accordingly; Chapter 3 illustrates the data collection and data analysis methods step by step. Chapter 4 discusses the analysis results and Chapter 5 summarizes the whole research with future research directions.

## Case Areas and Impact Factor Themes

### Sample Village Selection

**Figure 2:**
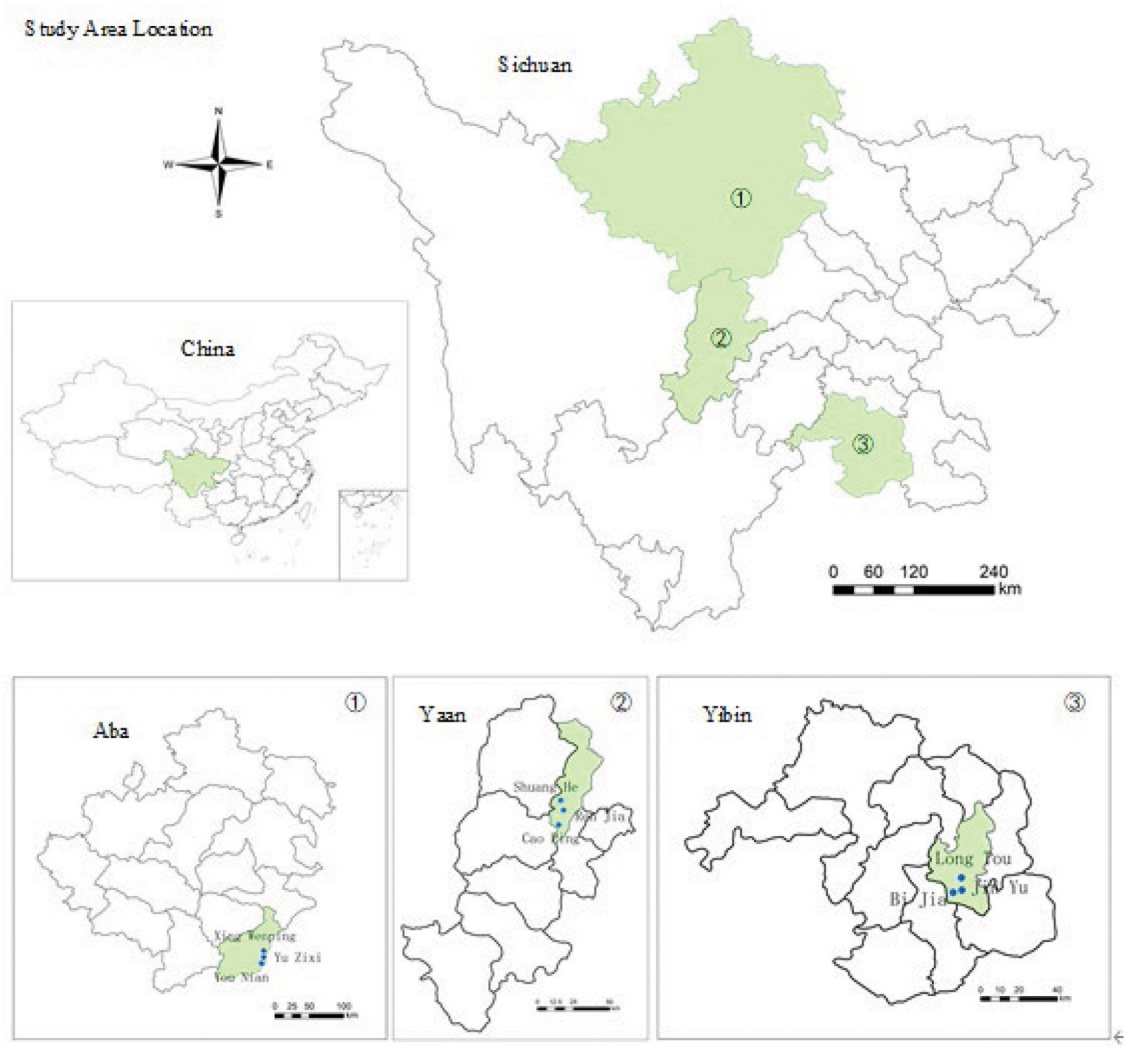
Sample villages distribution.

More than 80% of earthquakes in China are of a magnitude 5 or greater and occur in the countryside (34). Therefore, this study takes the rural residents who suffered the most from the three major earthquakes in Sichuan (Wenchuan 5.12 earthquake, Lushan 4.29 earthquake, and Changning 6.17 earthquake) as the research objects. A random sampling method was adopted to randomly select three villages in each of the rural areas severely affected by the three earthquakes. A total of nine villages have been selected as sample villages, namely Xing Wenping village, Oil Mill village, Yuzixi village in Wenchuan; Caocao village, Renjia village, Shuanghe village in Lushan; Bijia village, Goldfish village, Dragon village in Changning. The location distribution of sample villages is shown in Figure 2. These villages' contexts are used to combine with literature to identify four themes of impacting factors in Section Influencing Factor Themes.

### Influencing Factor Themes

Based on the literature review, we have identified four themes for grouping influencing factors on local residents' post-disaster resettlement choices. The data for all the factors under each theme is gathered for further analysis.

#### Social Demographic Factors

Existing studies have shown that respondents' social demographic background can significantly affect people's willingness to relocate after an earthquake (35, 36). Social demographic factors can be divided into personal factors and family factors. Personal factors include gender, age, household registration type, education level, job status, and current residence duration. And family factors include family type, the number of migrant workers, the main source of income, whether there is a motorcycle (scooter), and the number of motorcycles (scooters).

#### Residential Factors

Residential factors are mainly considered from the perspective of residents' living conditions. Existing literature shows that the condition of houses, such as the year of completion, has an impact on the selection of post-disaster resettlement mode (25). Therefore, this article also considers the factors like the year of the completion of the current house, whether the quality inspection has been carried out after the building is finished, and whether there is a shelter (open space) in the location. At the same time, the daily destination accessibility of local residents is also added as it significantly affects residents' willingness to choose among post-disaster resettlement modes (26).

#### Previous Recovery Experience Factor

Existing studies have shown that post-earthquake resettlement experience can affect their resettlement choice behavior again (23). Liu et al. (29) have studied the satisfaction of residents under the two post-disaster resettlement modes, namely the *in-situ* resettlement and the reallocated resettlement. The research shows that the overall satisfaction of reallocated resettlement is higher than that of *in-situ* resettlement. The same study group has also evaluated the satisfaction level of the previous recovery experience. In addition, in terms of neighborhood relations, family relations, and other home-related factors, the effect of *in-situ* resettlement are much better than that of reallocated resettlement (29). This study considers the impact of resettlement mode after the last earthquake experience as another factor theme.

#### Built Environment Perception Factors

Residents' perception of local risks may have an impact on their choices of resettlement modes. In the face of potential disasters, some people will make migration decisions, while others are unwilling to migrate for various reasons (33). Based on this, this study includes the local residents' perception of the built environment and explores their impacts on the choice made. There are many possible factors in this theme and therefore it is needed to find the most relevant ones for further analysis.

## Data Collection and Analysis Methods

According to the identified themes of impacting factors, questionnaires have been developed and validated by experts. Trained surveyors have been recruited and fieldwork has been performed to gather data from local residents using the questionnaires Section Data Collection. The descriptive statistics have been illustrated in Section Descriptive Statistics. With the collected data, we first have explored the most important factors for the built environment factor theme, using exploratory factor analysis. Based on the exploratory analysis, two binary logistic regression models have been constructed to identify the important factors under all themes that influence local residents' resettlement choices Section Binary Logistic Regression Analysis.

### Data Collection

To collect valid data, the research team has recruited 18 experienced researchers, including two faculty members, 14 graduate students, and two undergraduate students. The researchers are all from rural areas, including 16 from rural Sichuan. The surveyors all have the experience of a face-to-face questionnaire survey in villages and households. They are familiar with the rural environment and can effectively communicate with rural residents. Before the study, all 18 researchers received professional training in etiquette, questioning techniques, and the logical consistency of questions. In addition, the questionnaire was explained and discussed. If there are questions raised by the researchers on the questionnaire, the questionnaire is modified to address the questions. Investigators carried 10–20 questionnaires to one sample village for a preliminary survey. After the preliminary survey, researchers have adjusted the survey plan accordingly. By ensuring all issues resolved, a formal survey is finally conducted. The survey was conducted between January 5, 2020, and January 10, 2020. The households have been selected by random sampling. If the households are not willing to participate in the questionnaire survey, the next household will be randomly selected. All the personal sensitive data is anonymized and all the respondents have agreed to participate.

### Descriptive Statistics

The research group has distributed and assisted in filling out 900 questionnaires face-to-face, and finally completed 688 questionnaires effectively. After removing the questionnaires with too much missing content or lack of untruthful answers, 663 valid questionnaires have been obtained, with an effective rate of 96.37%. The distance from the epicenter of various villages and the number of valid questionnaires are shown in Table 1.

**Table 1:** Distance to epicenter and number of valid questionnaires.

**Sample villages**		**Distance from village center to epicenter (km)**	**Number of valid questionnaires**	**Number of households in the village**	**Total number of villages**
Wen Chuan	Xing Wenping	11^a^	74	200	800
	Yu Zixi	1.8	68	400	1,200
	You Nian	8.2	62	128	307
Lu Shan	Shuang He	16	97	424	1,500
	Ren Jia	9.4	85	1,090	3,346
	Cao Ping	4.3	68	854	3,704
Changning	Long Tou	6.1	86	340	1,000
	Jin Yu	0.1	71	390	1,200
	Bi Jia	1.1	52	210	600

a *This distance is the driving distance measured by Baidu navigation application*.

According to the statistics of 663 valid questionnaires, 404 people (60.94%) chose to settle in the original place, and the rest 259 people (39.06%) chose to settle in other places; 464 people (69.98%) chose centralized resettlement, while the rest 199 people (30.02%) chose scattered resettlement.

#### Social Demographic Theme

The basic information of the interviewees is shown in Table 2. According to the survey data, more women have been surveyed than men, which can be explained by the fact that male laborers go out to work, while females stay at home to deal with housework. Respondents over 40 years old accounted for 72.70% of the total. The employment situation in and around the surveyed villages cannot meet the needs of young people, so more of them choose to work or study elsewhere. The proportion of rural registered permanent residents is 85.37%, and some residents in the rural-urban fringe have changed to urban registered permanent residents, which indicates that the nature of rural China is gradually changing. The majority of people have had primary or junior high school education. The main types of personal income are farming and working outside. The number of years of living in the current place is generally long. The main types of families are parents and children living together and three generations living under the same roof. 58.82% of families have means of transportation for a short distance.

**Table 2:** Social demographic theme.

**Personal factor**		**Number**	**Proportion**	**Family property**		**Number**	**Proportion**
Gender	Male	303	45.70%	Homestyle	live alone	31	4.68%
	Female	360	54.30%		Two people live together	103	15.54%
age	(0, 20) year	28	4.22%		Parents live with their children	230	34.69%
	(20–30) year	75	11.31%		Of three generations under one roof	261	39.37%
	(30, 40) year	78	11.76%		Of four generations under one roof	27	4.07%
	(40, 50) year	146	22.02%		Other	11	1.66%
	(50, 60) year	175	26.40%	Migrant workers	0 people	264	39.82%
	(60, ∞) year	161	24.28%		1 people	164	24.74%
Type of registered permanent residence	Rural household registration	566	85.37%		2 people	179	27.00%
	Urban registration	97	14.63%		3 people	30	4.52%
Schooling	Nothing	78	11.76%		4 or more people	26	3.92%
	Primary school	255	38.46%	The main source of income	Agricultural industry	61	9.20%
	Junior high school	220	33.18%		Out-migrantion for work	398	60.03%
	Senior high school	71	10.71%		Shop management	98	14.78%
	Bachelor degree or above	39	5.88%		Domestic workshop	6	0.90%
working condition	No job	248	37.41%		House for rent	3	0.45%
	Farming	103	15.54%		Agritainment	4	0.60%
	Out-migrantion for work	137	20.66%		Other	93	14.03%
	In business	83	12.52%	Motorcycle or not	No	273	41.18%
	Governmental service	17	2.56%		Yes	390	58.82%
	Other	75	11.31%	Number of motorcycles	0	273	41.18%
Length of residence in current place	(0, 10) year	165	24.89%		1	346	52.19%
	(10, 20) year	109	16.44%		2	40	6.03%
	(20, 30) year	105	15.84%		3	3	0.45%
	(30, 40) year	70	10.56%		4 or more	1	0.15%
	(40, ∞) year	214	32.28%				

#### Residential Factor Theme

The variables of the current residential building year are classified as follows: “before 2000 (1),” “2000-2004 (2),” “2005-2009 (3),” “2010-2014 (4),” and “2015-2020 (5).” Whether the quality complies with the requirements variables are classified as “none (0)” and “yes (1)” after the building is built. Whether there is shelter (open space) on the location is classified as “none (0)” and “yes (1).” Various distance variables are classified as: “(0, 1) KM (1),” “(1, 2)KM (2),” “(2, 3) KM (3),” “(3, 4) KM (4),” “(4, up) KM (5).” The researchers have used Ove software to locate points on sites and measure distances from homes to the nearest school, health center (hospital), county center, and bus stops. After preliminary statistical analysis, the average value of these variables and the data sources are shown in Table 3.

**Table 3:** Residential condition items and sources.

**Items**	**Average (KM)**	**Source**
The year of construction of the present residence.	3.62	(25)
Is there any quality inspection after the building is built?	0.44	(37)
Is there a shelter (open space)?	0.89	(38)
The distance from the present residence to the nearest school.	1.70	(39)
The distance from the present residence to the nearest health center (hospital).	1.71	(39)
The distance from the present residence to the center of the county.	3.63	(39)
The distance from the present residence to the nearest bus station.	1.52	(39)

#### Previous Recovery Experience Theme

The two resettlement modes are classified as “in-situ resettlement (0)” and “reallocated resettlement 3 (1)” or “centralized resettlement (0)” and “scattered resettlement (1).” The quantified mean of statistical data and their sources are shown in Table 4.

**Table 4:** Previous recovery experiences items and sources.

**Items**	**AVG**	**Source**
The last time after the earthquake to choose the *in-situ* resettlement or reallocated resettlement? / The last time after the earthquake to choose centralized resettlement or scattered resettlement?	0.34/0.33	Authors

#### Built Environment Perception Theme

Combined with the existing research, in the design of the built environment perception questionnaire, the residents' satisfaction with their own environment and their risk perception in the future will have an impact on the expected results. The questionnaire asked 27 questions about the perception of the built environment. Using Likert Scale 5, 1 means “totally disagree” and 5 means “totally agree.” Respondents need to evaluate these 27 statements according to their own judgment. In order to determine important factors, factor analysis has been used to reduce the dimension of built environment perception factors for later analysis.

Factor analysis was first proposed by Chales Spearman in 1904 (37). Factor analysis is a statistical method to simplify and analyze high-dimensional data. Its principle is dimensionality reduction, which condenses complex variables into a few factors (40). This method causes the least information loss and can ensure the integrity of original information to the maximum extent (41). Since under the built environment theme, there are many factors involved, we have applied factor analysis to explore the most significant factors for further logistic regression analysis. The general form of factor analysis is:


(1) $$\begin{array}{l} x_{i}=a_{i1}F_{1}+a_{i2}F_{2}+\ldots +a_{in}F_{n}+\varepsilon_{i}\left(i=1,2,\ldots ,p\right) \end{array}$$

*Where* ***a_ij_*** *is the correlation coefficient between the common factor* ***F_i_*** *and the variable* ***x_i_*** *and* ***ε_i_*** *is the special factor, representing the influencing factors other than the common factor* (37).

Before the factor analysis, Kjeldahl Meyer Olgin (KMO) and Bartlett tests are carried out. In general, if the KMO value is >0.70 and the *p* value of the Bartlett variance homogeneity test is <0.05, the data is considered suitable for factor analysis (37). The test results are shown in Table 5. The KMO value is 0.879 and the *p* value is 0.000. The testing effect is significant, indicating that there is a certain correlation, which is suitable for factor analysis.

**Table 5:** KMO and Bartlett's test results.

**KMO and Bartlett test**		**Built environment perception**
KMO Sampling suitability quantity		0.879
Bartlett test of sphericity	Approximate chi square	9,632.906
	freedom	351
	Significance	0.000

The results of factor analysis are shown in Table 6. In general, if the contribution rate of accumulation variance of each factor reaches more than 60%, the scale is considered to have good validity. The total variance interpretation reaches 69.933%, that is, the factor analysis can explain 69.933% of the variables, and the effect of factor analysis is good. The closer the load coefficient is to 1, the more relevant the index is to the common factor. Generally, we can ignore load coefficients <0.4. After removing the factors whose factor load is <0.4, seven main factors are obtained.

**Table 6:** EFA results of BE perception: factor component matrix.

	**Component**							**Source**
	**Satisfaction with current housing**	**Satisfaction with current residence**	**Sense of security and belonging to the current residence**	**Satisfaction with government subsidies**	**Impact of the last earthquake**	**Interpersonal relationship**	**Risk perception of earthquake recurrence**	
	1	2	3	4	5	6	7	
Satisfaction with house power supply	0.730							(28–30)
Satisfaction with house gas supply	0.733							(28–30)
Satisfaction with house drainage	0.740							(28–30)
Satisfaction with housing structure	0.812							(28–30)
Satisfaction with housing area	0.778							(28–30)
Satisfaction with house orientation and daylighting	0.788							(28–30)
Satisfaction with the quality of life in the place of residence		0.589						(28–30)
Satisfaction with residence economy		0.701						(28–30)
Satisfaction with the ecological environment of the residence		0.425						(28–30)
Satisfaction with the educational environment of the residence		0.737						(28–30)
Satisfaction with medical conditions in residence		0.762						(28–30)
Satisfaction with residential infrastructure		0.753						(28–30)
Satisfaction with employment opportunities in residence		0.815						(28–30)
Sense of belonging to the place of residence			0.803					(29)
Security of place of residence			0.788					(29)
Road connections to safe locations			0.693					(28)
Adaptability to local customs			0.556					(30, 42)
Satisfaction with the amount of government subsidies				0.860				(23, 29)
Satisfaction with government subsidies				0.895				(23, 29)
Satisfaction with the transparency of government subsidy information				0.848				(23, 29)
The degree of damage to the building structure caused by the last earthquake					0.893			(30, 42)
The damage degree of the last earthquake to the road					0.821			(30, 42)
The impact of the last earthquake on you and your family					0.850			(43)
Neighborhood relations						0.858		(29)
Family relations						0.853		(29)
There is a great possibility of another destructive earthquake							0.829	(32, 33)
When another destructive earthquake occurred, the family was greatly affected							0.737	(32, 33)
Inclusion statistics								
Characteristic value	4.024	3.789	2.846	2.545	2.318	1.991	1.369	
Percentage variance	14.904	14.032	10.542	9.426	8.586	7.374	5.069	
Cumulative variance percentage	14.904	28.936	39.478	48.904	57.490	64.864	69.933	

*Extraction method: principal component analysis.*

*Rotation method: Kaiser standardized orthogonal rotation method.*

*The rotation has converged after 6 iterations*.

### Binary Logistic Regression Analysis

To understand all the factors identified that impact resettlement mode choices, binary logistic regression analysis has been performed. The binary Logistic regression model does not require the distribution of explanatory variables, nor does it require a linear relationship between explanatory variables and the explained variables. This makes it suitable for analyzing problems with classified variables. In the general regression model, the dependent variable of the function is usually an interval variable, and the dependent variable should meet the assumption conditions of normal distribution. Logistic regression is different from general regression models. The purpose of logistic regression is to predict the probability of each classification of a certain classification variable, so the dependent variable must be a classification variable, and logistic regression has no special requirements for independent variables, which can be interval variables, categorical variables, or a mixture of the two variables. Meanwhile, according to the number of dependent variable values, the logistic regression model is further divided into a binary logistic regression model and a multivariate logistic regression model. In the multivariate logistic regression model, the dependent variable can take multiple values. In the binary logistic regression model, the dependent variable is a binary value, set as ***Y*** and follows a binomial distribution with values of 0 and 1, and the independent variables are ***X_1_, X_2_***, …, ***X_n_***. The binary logistic regression model corresponding to independent variables is:


(2) $$\begin{array}{l} P\left(Y=1\right)=\frac{EXP\left(\beta_{0}+\beta_{1}X_{1}+\beta_{2}X_{2}+\ldots +\beta_{n}X_{m}\right)}{1+EXP\left(\beta_{0}+\beta_{1}X_{1}+\beta_{2}X_{2}+\ldots +\beta_{n}X_{n}\right)} \end{array}$$

(3) $$\begin{array}{l} P\left(Y=0\right)=\frac{1}{1+EXP\left(\beta_{0}+\beta_{1}X_{1}+\beta_{2}X_{2}+\ldots +\beta_{n}X_{n}\right)} \end{array}$$

(4) $$\text{logitP}\left(\text{Y}=1\right)=\beta_{0}+\beta_{1}\;{\text{X}}_{1}+\beta_{2}{\text{X}}_{2}+\ldots +\beta_{n}{\text{X}}_{n}$$

Similar to the linear regression model, ***β_0_*** is the constant term (or intercept), and ***β_i_*** is the partial regression coefficient corresponding to ***X_i_(i = 1, 2***, …, ***m***) (43).

For our study, binary logistic regression analysis has been conducted as we have only two options for resettlement modes for each model.

Taking all the above factors as independent variables, selecting set [*in-situ* resettlement (0), reallocated resettlement (1)], [centralized resettlement (0), scattered resettlement (1)] as dependent variables, binary logistic regression analysis has been performed using SPSS 20.0 software. The results of binary logistic regression are shown in Table 7. And the model fitting results show that the chi-square scores are 9.188 and 8.574 with degrees of freedom of 8, and the Cox-Snell R2 values are 0.317 and 0.330, respectively. The fitting effect of the regression model is good, and the data can be further correlated. When there are many independent factors in the actual problem, there may be a certain correlation between two or more independent variables, which is called multicollinearity. When the collinearity trend of independent variables is very obvious, it will seriously affect the fitting of the model (43). In this study, variance expansion factor (VIF) has been used to test multicollinearity. The greater the variance expansion factor, the stronger the multicollinearity. When the VIF value is >10, it is considered that there is strong multicollinearity between variables and it is unacceptable (44). The VIF values of the independent variables in this research are <10 (see Table 7), indicating that there is no multicollinearity between the independent variables.

**Table 7:** Results of binary logistic regression analysis.

**Variable**	***In-situ*** **resettlement (0)**			**Centralized resettlement (0)**		
	**Reallocated resettlement (1)**			**Scattered resettlement (1)**		
	**B**	**Significance**	**VIF**	**B**	**Significance**	**VIF**
**Personal attributes**						
Gender	−0.262	0.229	1.117	0.242	0.306	1.106
Age	−0.360	0.000	2.031	−0.220	0.043	2.019
Length of current residence	−0.214	0.005	1.652	−0.170	0.043	1.519
Account type	−0.203	0.480	1.101	−0.554	0.084	1.101
Education level	0.372	0.007	1.881	0.209	0.164	1.897
Working condition (no working)		0.320	1.202		0.369	1.209
Working conditions (farming)	−0.651	0.076		−0.187	0.649	
Working conditions (working outside)	−0.221	0.621		−0.147	0.763	
Working conditions (business)	−0.160	0.684		−0.106	0.817	
Working conditions (government services)	−0.629	0.204		0.712	0.173	
Working conditions (others)	−0.010	0.988		−0.956	0.258	
**Family attributes**						
Main source of income (migrant workers)		0.149	1.123		0.986	1.123
Main source of income (agricultural production)	0.577	0.237		0.049	0.917	
Main source of income (shop operation)	0.841	0.018		−0.074	0.838	
Main source of income (family workshop)	0.745	0.114		−0.389	0.419	
Main source of income (house rental)	3.044	0.019		−0.595	0.620	
Main source of income (farmhouse)	−19.191	0.999		−21.267	0.999	
Main source of income (other)	0.898	0.508		−18.324	0.999	
Family type	−0.347	0.002	1.082	0.044	0.704	1.089
Number of migrant workers	0.203	0.038	1.116	−0.119	0.285	1.117
Is there a motorcycle (battery car)	0.368	0.389	4.151	1.332	0.003	4.146
Number of motorcycles (battery cars)	0.324	0.340	4.083	1.161	0.001	4.083
**Residential properties**						
Year of completion of current residence	0.294	0.006	1.166	−0.065	0.531	1.163
Is there any quality acceptance after the house is built	−0.159	0.494	1.308	−0.640	0.013	1.335
Is there a shelter (open space) in the location	0.228	0.520	1.200	−0.689	0.058	1.176
Distance to nearest school	0.596	0.032	8.323	0.362	0.225	8.313
Distance to nearest health center (hospital)	0.508	0.040	7.687	0.486	0.058	7.710
Distance to town center	0.031	0.695	1.473	0.112	0.181	1.516
Distance to nearest bus stop (bus stop)	0.268	0.054	1.777	0.051	0.747	1.728
**Past recovery experience**						
After the last earthquake, choose the *in-situ* resettlement or reallocated resettlement./centralized resettlement or scattered resettlement.	−1.853	0.000	1.399	−2.358	0.000	1.361
**Built environment perception**						
Satisfaction with current housing	0.057	0.596	1.164	0.036	0.761	1.180
Satisfaction with current residence	−0.283	0.009	1.180	0.170	0.152	1.172
Sense of security and belonging to the place of residence	−0.020	0.841	1.088	−0.223	0.046	1.078
Satisfaction with government subsidies	0.002	0.985	1.055	−0.085	0.476	1.056
Damage of houses and roads and its impact on families	0.268	0.028	1.355	−0.408	0.001	1.315
interpersonal relationship	0.113	0.268	1.077	0.071	0.539	1.069
Risk perception	0.205	0.066	1.158	−0.494	0.000	1.146
Constant	−1.575	0.192		0.083	0.949	

## Results

### Social Demographic Factor Theme Results

For personal factors, gender has no obvious impact on the resettlement modes choice. The older the residents are and the longer they live in their current residence, the more inclined they are to choose the centralized *in-situ* resettlement. The reason may be the strong habit and dependence on local facilities, and this is consistent with the study of Lu et al. (23). The more educated (B = 0.372, *p* = 0.007) the residents are, the more they want to resettle in other places. In the study of Zorrilla and Sandberg, it is also found that residents with higher education are also more likely to choose to migrate outward under natural disasters (45), which is consistent with the results of this study. Residents with rural household registration account type (B = −0.554, *p* = 0.084) tend to choose scattered resettlement. Other research supports this finding as well (21).

Compared with families whose main source of income is migrant workers, people who earn money by running shops and renting houses are more inclined to choose reallocated resettlement. Families with a large number of migrant workers (*B* = 0.203, *p* = 0.038) prefer to relocate to other places, where they can find better employment opportunities. The object of this study is rural China and the income in rural areas is generally not high. Young villagers prefer to seek better employment opportunities in other places because there are few employment opportunities in rural areas and the employment scope is narrow. If this trend continues, the rural area will be with more elderly and children which makes them more fragile in disasters; therefore, a better designed centralized settlement that can attract these residents to relocate might be preferred. On the other hand, in the future, the development of characteristic industries in rural areas and surrounding areas might enable young people to obtain a certain income without going out to work, which will affect the choice of post-earthquake resettlement models for rural residents. This is consistent with the proposal of Kniveton et al. that different employment opportunities will have an impact on residents' choice of relocation (46). Families living together for several generations (B = −0.347, *p* = 0.002) in the local area prefer to choose the *in-situ* resettlement, possibly because of their strong attachment to the local area. The existence and number of motorcycles significantly affect whether residents choose centralized resettlement or scattered resettlement. Families with more motorcycles (B = 1.161, *p* = 0.001)prefer scattered resettlement. Similar studies (47) show consistent results as well.

### Residential Factor Theme Results

In the residential factor theme, the completion year and accessibility of the current housing significantly affect the residents' choice of *in-situ* resettlement or reallocated resettlement. To be more specific, the earlier the current residence (B = 0.294, *p* = 0.006) is built, the weaker their intention is to move, which is consistent with the analysis in the personal factor theme. That is the longer the residence time, the more desire for residents to choose the *in-situ* resettlement. The closer the residence is to the nearest school (B = 0.596, *p* = 0.032), health center (hospital) (B = 0.508, *p* = 0.040) and bus stop (B = 0.268, *p* = 0.054), the more likely it is for them to choose the *in-situ* resettlement. In transportation-related research, the choice behavior of residence or work place is usually related to public transport accessibility, travel cost, travel mode, traffic congestion, and departure time (48, 49). In general, the attractiveness of a place increases with the accessibility of the workplace and daily life (26). This supports the findings in this research as well. Furthermore, whether the quality acceptance is carried out after the house is built (*B* = −0.640, *p* = 0.013), whether there is a shelter (open land))(*B* = −0.689, *p* = 0.058) on the location and the distance from the hospital (B = 0.486, *p* = 0.058) near the residence significantly affect the residents' choice between centralized resettlement and scattered resettlement. This finding aligns with the conclusion of Lindell et al. (32) that residents prefer to choose centralized resettlement when they think their residential conditions are relatively safe.

### Previous Recovery Experience Theme Results

Based on the valid 464 questionnaire results answering questions in this theme, the resettlement experience after the last earthquake significantly affects the resettlement mode choice made in the future. It can be seen from Table 7 that when the resettlement mode selected after the last earthquake is *in-situ*, residents may prefer to choose non-local resettlement in the future resettlement (B = −1.853, *p* = 0.000). For people with the centralized resettlement (B = −2.358, *p* = 0.000) after the last earthquake, may be more willing to choose scattered resettlement in the future. This shows that residents prefer to choose the opposite resettlement mode in the future. This finding indicates that in the rural areas of Sichuan, there has been not effective and satisfactory resettlement planning which could result in the consistent choice for desired resettlement from the local residents in the future. This finding also aligns with another survey made by the same group of researchers to evaluate the satisfaction levels of residents' previous resettlement experience and the results show that residents are not happy with their previous experience. Therefore, this study is in needs of a better design of future resettlement based on residents' preferences, using empirical data.

### Built Environment Perception Theme Results

Overall, the impact of built environment perception on the choice of villagers' -post-disaster resettlement mode is limited. Among the perceived variables of the built environment, satisfaction with the current residence has a significant impact on residents' choice of *in-situ* resettlement or reallocated resettlement. Residents who are satisfied with their current residence (B = −0.283, *p* = 0.009) are more likely to choose the *in-situ* resettlement. In fact, environmental factors have been proved to be an important consideration in site selection decision-making, and people prefer to live in a place with a good environment (50, 51), which is consistent with the results of this study. The more sense of security and belonging to the place of residence, the more residents are willing to choose centralized resettlement (B = −0.223, p = 0.046). The more serious the damage to houses and roads and the impact on families (B = −0.408, *p* = 0.001), the higher the risk perception (B = −0.494, *p* = 0.000), the more residents prefer centralized resettlement. A study in Vietnam shows similar results (52). We can also see that there are some factors identified in the literature that do not have an impact on resident's post-disaster resettlement choices in rural Sichuan. Therefore, it indicates that it is needed to follow context-specific data analysis for better village design in the post-disaster era.

## Discussion

This research filled in the current research gaps from three perspectives: research context and analysis angle; research data and method; research results.

Regarding the research context and analysis angle, previous studies on post-disaster resettlement behavior mostly focus on urban areas in developed countries and analyze them from the perspective of the government, while few studies have focused on resettlement behavior in developing countries and poverty-stricken areas, from the household's perspective. Facing the requirements of human-centric planning, it is, therefore, essential to understand residents' willingness to help the government to design better sustainable resettlement. This study fills the gap in this regard. The choice of rural post-disaster resettlement mode is analyzed to further understand the needs of residents.

Due to the nature of the frequent earthquake occurrence in Sichuan, this study considers not only the previously identified variables for their impact on the choice of residents' post-disaster resettlement mode but also adds the variables of the last post-disaster resettlement pattern choice and experience. This means that the research objects are rural residents who have experienced an earthquake. Therefore, this research provides insights by following up with residents under a sequence of disasters circumstances to offer a more solid and systematic understanding of the situation instead of a single occurrence.

From the data and method perspective, this article selects the rural areas where the three major earthquakes occurred in Sichuan Province recent and select nine sample villages in a reasonable way for investigation. Due to the inaccuracy and low granularity of rural area facilities data from official sources, we have combined field survey with geo-location coding so that these facilities' locational data can be more up-to-date and accurate. This fills in the data gap not only in the rural resettlement planning field but also in other fields like rural infrastructure design, facility management, and so on.

Regarding the research results, among the residential properties, the earlier the existing residential building was built, the weaker the residents' relocation intention is. Generally speaking, the longer the house exists, the worse its function will be. When the existing house becomes less able to meet the needs of the residents, it would enlarge the residents' willingness to move to a certain extent (25). However, this research shows the opposite. This could be potentially explained by that the earlier the construction year of the existing house, the stronger the dependence on the locality, therefore the weaker the intention to move. One other possible reason could be due to the low-income level that they are not able to move elsewhere even with the resettlement choice offered. The result of this factor triggers further investigation needed to fully understand the reasons.

The main limitation of this study is that in terms of social demographic variables, this study does not consider wealth but those with more financial means may choose differently. This also aligns with the previous potential reasons why our research results are not consistent with the other research regarding relocation intentions. However, we do have selected motorbike possession as one of the variables which can reflect some extent the wealth of residents. In terms of space, this study does not analyze the specific relocation orientation, resulting in no an in-depth analysis of the influencing factors, and it only preliminary discusses the relationship between rural residents' relocation intention and its influencing factors. In future research, we shall consider the wealth factor and analyze the difference in residents' choice of post-disaster resettlement modes. we could also analyze the specific relocation orientation of residents with different relocation intentions.

## Conclusions

Facing severe earthquake threats in Rural China, the government needs to design a built environment for better resettlement in a human-centric and resilient way to improve quality of life and public health. The current studies focus mainly on other disasters than earthquakes in developed countries and analyze multiple factors in a non-systematic way. Therefore, the main purpose of this research is to explore the influencing factors under different themes on the resettlement mode choices made in rural China, using empirical data and systematic analysis.

We have chosen case study areas first and then identified four important themes affecting post-earthquake resettlement mode choices based on literature and the context in rural China. Questionnaires are developed accordingly and used for gathering data for these four themes' factors. For reducing factor numbers in themes, factor analysis has been applied. For analyzing all the factors in different themes, two binary logistic regression models with identified factors are built.

Through investigation and research, it is found that the factors affecting the choice of residents' resettlement modes are context-specific. Not all the identified factors have significant impacts on each resettlement mode.

The systematic analysis of the main influencing factors provides a better understanding of the motivation of residents' choice of resettlement modes in Rural China, which can not only guide rural residents to reasonably choose resettlement modes but also place people in the center for post-disaster resettlement design (53). This analysis also provides a reference for the planning and construction of future resilient villages in disaster-prone areas. In addition, among the influencing factors of choosing *in-situ* resettlement or reallocated resettlement, the negative correlation of satisfaction with the current residence is obvious, indicating that satisfaction with the current residence is still the main factor causing residents' migration. Therefore, in the overall construction and planning of the village in the future, residents' satisfaction should be put at the center. Possible actions could be: the traffic roads in the village should be actively improved and the construction of infrastructure and other public service facilities in the village shall be carried out simultaneously with the residential construction. The local economy should be improved so that young people would stay in the village and take better care of the elderly and children, making them less vulnerable to facing disasters.

## Data Availability Statement

The original contributions presented in the study are included in the article/supplementary materials, further inquiries can be directed to the corresponding author.

## Ethics Statement

Ethical review and approval was not required for the study on human participants in accordance with the local legislation and institutional requirements. The patients/participants provided their written informed consent to participate in this study.

## Author Contributions

LZ, SZ, YA, and YW: conceptualization, methodology, and data curation. LZ, SZ, and YW: software. LZ, SZ, YA, YW, and TW: formal analysis. LZ, SZ, YW, and TW: resources and writing—original draft preparation. JZ, YC, and TW: writing—revision. LZ, YA, YW, and TW: supervision. All authors have read and agreed to the published version of the manuscript.

## Funding

The article processing costs are funded by the Delft University of Technology. This study is also supported by the National Natural Science Foundation of China (72171028), Sichuan Rural Community Governance Research Center (SQZL2021A01 and SQZL2021B03), Sichuan Disaster Economics Research Center (ZHJJ2021-YB004), Meteorological Disaster Prediction and Emergency Management Research Center (ZHYJ21-YB06), Sichuan Rural Development Research Center (CR2101), Regional Public Management Information Research Center (QGXH21-02), Open Foundation of the Research Center for Human Geography of Tibetan Plateau and Its Eastern Slope (Chengdu University of Technology) (RWDL2021-ZD001), Energy and Environmental Policy Research Center, Chengdu University of Technology (CEE2021-ZD02), and Chengdu Philosophy and Social Science research Base-Chengdu Park Urban Demonstration Area Construction Research Center project (GYCS2021-YB001), and 2020 Sichuan Province Applied Basic Research Program Project (NO. 2020YJ0364).

## Conflict of Interest

The authors declare that the research was conducted in the absence of any commercial or financial relationships that could be construed as a potential conflict of interest.

## Publisher's Note

All claims expressed in this article are solely those of the authors and do not necessarily represent those of their affiliated organizations, or those of the publisher, the editors and the reviewers. Any product that may be evaluated in this article, or claim that may be made by its manufacturer, is not guaranteed or endorsed by the publisher.

## References

[1] Guo S Liu S Peng L Wang H. The impact of severe natural disasters on the livelihoods of farmers in mountainous areas: a case study of Qingping Township, Mianzhu City. Natural Hazards. (2014) 73:1679–96. 10.1007/s11069-014-1165-9

[2] Liu Y. Wenchuan earthquake in Sichuan and national earthquake relief. Chin Geol Educ. (2008) 16–20. 10.16244/j.cnki.1006-9372.2008.02.006

[3] Xu D Yong Z Deng X Liu Y Huang K. Financial preparation, disaster experience, and disaster risk perception of rural households in earthquake-stricken areas: evidence from the Wenchuan and Lushan Earthquakes in China's Sichuan Province. Int J Environ Res Public Health. (2019) 16:3345. 10.3390/ijerph16183345 31514264 PMC6765853

[4] China's National Bureau of Statistics. China Yearbook of Household Survey in 2019. Beijing, China: China Statistical Press (2020).

[5] Yang S. Study on migration of affected population in wenchuan earthquake area. Soc Sci Res. (2009) 1–7.

[6] Alexander D Davis I. Recovery From Disaster. New York, NY: Routledge (2015).

[7] Regulations on post wenchuan earthquake recovery and reconstruction. China Disaster Reduct. (2008) 60–4.

[8] Jha AK Barenstein JD Phelps PM Pittet Sena S. Safer Homes, Stronger Communities : A Handbook for Reconstructing After Natural Disasters. Washington, DC: World Bank Publications (2010). pp. 1–370.

[9] Iuchi K. Redefining a place to live: Decisions, planning processes, and outcomes of resettlement after disasters. University of Illinois at Urbana-Champaign (2010).

[10] Alaci D. Regulating urbanisation in Sub-Saharan Africa through cluster settlements:lessons for urban managers in Ethiopia. Theor Empir Res Urban Manag. (2010) 5:20–34.

[11] Yi P Shen L Tan C Tan D Wang H. Critical determinant factors (CDFs) for developing concentrated rural settlement in post-disaster reconstruction: a China study. Nat Hazards. (2012) 66:355–73. 10.1007/s11069-012-0488-7

[12] Yang G Shen F Chen W Shanshan M. Types and transect differentiation of external forms of Chinese traditional villages. J Hengyang Norm Univ. (2017) 38:8–12. 10.13914/j.cnki.cn43-1453/z.2017.03.002

[13] Durage SW Kattan L Wirasinghe SC Ruwanpura JY. Evacuation behaviour of households and drivers during a tornado. Nat Hazards. (2013) 71:1495–517. 10.1007/s11069-013-0958-6

[14] Lazo JK Bostrom A Morss RE Demuth JL Lazrus H. Factors Affecting Hurricane Evacuation Intentions. Risk Anal. (2015) 35:1837–57. 10.1111/risa.12407 26299597

[15] Gat A Lmw B Ecj C Adm C Sjg A Cvp B. The role of individual well-being in risk perception and evacuation for chronic vs. acute natural hazards in Mexico. Appl Geogr. (2011) 31:700–11. 10.1016/j.apgeog.2010.12.008

[16] Xuan B. Fukushima nuclear disaster displacement: how far people moved and determinants of evacuation destinations. Int J Disaster Risk Reduct. (2019) 33:235–52. 10.1016/j.ijdrr.2018.10.009

[17] Bernadeth B Hector R Mongkut P Francis A. A household-level flood evacuation decision model in Quezon City, Philippines. Nat Hazards. (2015) 80:1539–61. 10.1007/s11069-015-2038-6

[18] Mortreux C Barnett J. Climate change, migration and adaptation in Funafuti, Tuvalu. Glob Environ Change. (2009) 19:105–12. 10.1016/j.gloenvcha.2008.09.006

[19] Huang S Lindell MK Prater CS Wu H Siebeneck LK. Household Evacuation Decision Making in Response to Hurricane Ike. Nat Hazards Rev. (2012) 13:283–96. 10.1061/(ASCE)NH.1527-6996.0000074

[20] Xu D Li P Liu S Su C Wang X. Influences of sense of place on farming households' relocation willingness in areas threatened by geological disasters: evidence from China. Int J Disaster Risk Sci. (2017) 8:16–32. 10.1007/s13753-017-0112-2

[21] Chen Y. Preliminary discussion on disaster and migration. J Catastrophol. (2009) 24:138–44. 10.1111/j.0954-6820.1952.tb04049.x

[22] Morrow-Jones HA Jones CR. Mobility due to natural disaster: theoretical considerations and preliminary analyses. Disasters. (1991) 15:126–32. 10.1111/j.1467-7717.1991.tb00441.x 20958718

[23] Lu Q Zhang J Rahman ABMS. Location choice behavior adapting to flood and cyclone hazards. Int J Disaster Risk Reduct. (2018) 27:189–98. 10.1016/j.ijdrr.2017.10.006

[24] Osterling JP. Peruvian disaster and the spontaneous relocation of some of its victims: Ancashino peasant migrants in Huayopampa. Mass Emerg. (1979) 4:117–20.

[25] Li J Li X. Analysis on influencing Factors of rural residents' willingness to move. Econ Geograp. (2008) 28:454–9. 10.15957/j.cnki(2008).03.014

[26] Eliasson J. The influence of accessibility on residential location. Adv Spat Sci. (2010) 20. 10.1007/978-3-642-12788-5_7

[27] Ao Y Yang D Chen C Wang Y. Exploring the effects of the rural built environment on household car ownership after controlling for preference and attitude: evidence from Sichuan, China. J Transp Geograp. (2019) 74:24–36. 10.1016/j.jtrangeo.2018.11.002

[28] Bhat CR Jessica G. A mixed spatially correlated logit model: formulation and application to residential choice modeling. Transp Res B Methodol. (2004) 38:147–68. 10.1016/S0191-2615(03)00005-5

[29] Liu H Zhang D Qu W Guo Z. Comparison study on two post-earthquake rehabilitation and reconstruction modes in China. Int J Disaster Risk Reduct. (2017) 23:109–18. 10.1016/j.ijdrr.2017.04.016

[30] Yi P Shen Q Shen L Chen L Zhao Y. A generic decision model for developing concentrated rural settlement in post-disaster reconstruction: a China study. Nat Hazards. (2013) 71:611–37. 10.1007/s11069-013-0924-3

[31] Wijegunarathna EE Wedawatta G Prasanna LJ Ingirige B. Long-term satisfaction of resettled communities:an assessment of physical performance of post-disaster housing. Procedia Eng. (2018) 212:1147–54. 10.1016/j.proeng.2018.01.148

[32] Lindell MK Perry RW. Household adjustment to earthquake hazard: a review of research. Environ Behav. (2000) 32:461–501. 10.1177/00139160021972621

[33] Wachinger G Renn O Begg C Kuhlicke G. The risk perception paradox–implications for 611governance and communication of natural hazards. Risk Anal. (2013) 33:1049–65. 10.1111/j.1539-6924.2012.01942.x 23278120

[34] Zhu Z Ha H Hu A Xu C Ji S Chen Y. Summary of China's rural earthquake safety policies and regulations. Recent Dev World Seismol. (2015) 3:3–19. 10.3969/j.issn.0235-4975.2015.03.003

[35] Anamaria B Andrew S Angang Z. Evaluating drivers of coastal relocation in Hurricane Sandy affected communities. Int J Disaster Risk Reduct. (2015) 13:215–28. 10.1016/j.ijdrr.2015.06.008

[36] Shah AA Ye J Shaw R Ullah R Ali M. Factors affecting flood-induced household vulnerability and health risks in Pakistan: the case of Khyber Pakhtunkhwa (KP) Province. Int J Disaster Risk Reduct. (2020) 42. 10.1016/j.ijdrr.2019.101341

[37] Ma X. Multicollinearity diagnosis method in linear regression equation and its empirical analysis. J Huazhong Agri Univ. (Soc Sci Edn). (2008) 78-81+85. 10.13300/j.cnki.hnwkxb.2008.02.005

[38] Shiwakoti N. Understanding differences in emergency escape and experimental pedestrian crowd egress through quantitative comparison. Int J Disaster Risk Reduct. (2016) 20:129–37. 10.1016/j.ijdrr.2016.11.002

[39] Jonas E. Residential Location Choice: Models and Applications. Pagliara F, Preston J, Simmonds D, eds. Springer: Berlin Heidelberg (2010). Pp. 137–64.

[40] Zeng W Yan S Tian J. Quality evaluation of new urbanization in Wuhan based on factor analysis. Stat Decis. (2019) 35:114–7.

[41] Zhang Y Yang H Ma K. Research on residents' happiness of Tonghua City based on factor analysis. Technol Wind. (2019) 232.

[42] Li Y Guo Y. Be proactive for better decisions: Predicting information seeking in the context of earthquake risk. Int J Disaster Risk Reduct. (2016) 19:75–83. 10.1016/j.ijdrr.2016.08.008

[43] Zhang J. Environmental hazards in the Chinese Public's eyes. Risk Anal. (1994) 14:163–7. 10.1111/j.1539-6924.1994.tb00041.x 8008925

[44] Wu S. Beijing: Tsinghua University Press (2014).

[45] Saldaña Z Sergio O Krister S. Impact of climate-related disasters on human migration in Mexico: a spatial model. Clim Change. (2009) 96:97–118. 10.1007/s10584-009-9577-3

[46] Kniveton D Smith C Wood S. Agent-based model simulations of future changes in migration flows for Burkina Faso. Glob Environ Change. (2011) 21:S34–40. 10.1016/j.gloenvcha.2011.09.006

[47] Bhat CR Jessica YG. A comprehensive analysis of built environment characteristics on household residential choice and auto ownership levels. Transp Res B Methodol. (2007) 41:506–26. 10.1016/j.trb.2005.12.005

[48] Arentze T Timmermans H. Congestion pricing scenarios and change of job or residential location: resultsof a stated adaptation experiment. J Transp Geograp. (2007) 15:56–61, 10.1016/j.jtrangeo.2006.02.013

[49] Nurlaela S Curtis C. Modeling household residential location choice and travel behavior and its relationship with public transport accessibility. Procedia Soc Behav Sci. (2012) 54:56–64. 10.1016/j.sbspro.2012.09.725

[50] Klaiber HA. Migration and household adaptation to climate: a review of empirical research. Energy Econ. (2014) 46:539–47. 10.1016/j.eneco.2014.04.001

[51] Tacoli C. Crisis or adaptation? Migration and climate change in a context of high mobility. Environ Urban. (2009) 21:513–25. 10.1177/0956247809342182

[52] Koubi V Spilker G Schaffer L Bernauer T. Environmental stressors and migration: evidence from Vietnam. World Dev. (2016) 79:197–210. 10.1016/j.worlddev.2015.11.016

[53] Maly E. Building back better with people centered housing recovery. Int J Disaster Risk Reduct. (2018) 29:84–93. 10.1016/j.ijdrr.2017.09.005

